# Incremental Prognostic Value of Fetal Doppler Beyond Biometry for Adverse Perinatal Outcomes in Suspected Fetal Growth Restriction: A Retrospective Cohort Study

**DOI:** 10.3390/biomedicines14102370

**Published:** 2026-10-17

**Authors:** Deniz Taşdemir, Murat Haksever, Selim Kandemir, Bekir Kahveci, Refaettin Şahin, Savaş Özdemir, Ramazan Adan, Cihan Yüksel, Ismet Hortu

**Affiliations:** 1 Department of Perinatology, Sanliurfa Training and Research Hospital, Sanliurfa 63250, Turkey; 2 Department of Gynecologic Oncology, Sanliurfa Training and Research Hospital, Sanliurfa 63250, Turkey; 3 Department of Obstetrics and Gynecology, Sanliurfa Training and Research Hospital, Sanliurfa 63250, Turkey; 4 Department of Obstetrics and Gynecology, Liv Hospital, Istinye University Faculty of Medicine, Istanbul 34010, Turkey; 5 Department of Obstetrics and Gynecology, Recep Tayyip Erdogan University Faculty of Medicine, Rize 53200, Turkey; 6 Department of Obstetrics and Gynecology, Cemil Taşcıoğlu City Hospital, University of Health Sciences, Istanbul 34384, Turkey; 7 Department of Obstetrics and Gynecology, Kastamonu Nefes Hospital, Kastamonu 37100, Turkey; 8 Department of Obstetrics and Gynecology, Ege University Faculty of Medicine, Izmir 35100, Turkey

**Keywords:** fetal growth restriction, small for gestational age, umbilical artery Doppler, cerebroplacental ratio, perinatal outcome, prognostic model

## Abstract

**Objective**: To explore the incremental prognostic information of fetal Doppler measurements beyond maternal and fetal biometric characteristics for adverse perinatal outcomes in pregnancies with suspected fetal smallness. **Methods**: This retrospective cohort study included 606 singleton pregnancies assessed for suspected or diagnosed fetal growth restriction. The principal analysis was restricted to 429 pregnancies with estimated fetal weight (EFW) or abdominal circumference (AC) below the 10th percentile at the index examination. A composite adverse perinatal outcome comprised five-minute Apgar score < 7, umbilical arterial pH < 7.10, neonatal intensive care unit (NICU) admission, respiratory support, neonatal death, or stillbirth. Baseline clinical–biometric, Doppler-integrated, and delivery-week sensitivity logistic models were assessed using stratified 10-fold out-of-fold predictions. Additional robustness analyses evaluated component frequencies, a hard-outcome sensitivity composite less directly influenced by clinical management, paired uncertainty in out-of-fold performance differences, and model performance across alternative cohort definitions. **Results**: Among 429 pregnancies in the principal cohort, 111 (25.9%) met the composite adverse perinatal outcome, and 206 (48.0%) had abnormal Doppler findings. Event rates were 85/206 (41.3%) among pregnancies with abnormal Doppler and 26/223 (11.7%) among those with normal Doppler. The baseline and Doppler-integrated out-of-fold areas under the receiver operating characteristic curve (AUCs) were 0.642 and 0.690, respectively; corresponding Brier scores were 0.183 and 0.174. Elevated umbilical artery pulsatility index with forward flow and absent or reversed end-diastolic flow (AEDF/REDF) were independently associated with the composite outcome, with adjusted odds ratios of 2.61 (95% confidence interval [CI], 1.58–4.32) and 6.60 (95% CI, 2.40–18.18), respectively, compared with normal umbilical artery flow. The adjusted estimate for the AEDF/REDF subgroup was imprecise because this subgroup included only 19 pregnancies. The paired out-of-fold AUC increment for the Doppler-integrated model was 0.049 (95% CI, 0.005–0.094); the corresponding Brier-score improvement was 0.009 (95% CI, −0.0004 to 0.0179). Sensitivity analyses supported a modest incremental contribution but did not establish the superiority of the integrated model over alternatives based on the cerebroplacental ratio (CPR) or umbilical artery (UA) Doppler. **Conclusions**: In pregnancies with suspected fetal smallness defined by EFW or AC below the 10th percentile, fetal Doppler assessment provided modest incremental prognostic information beyond maternal and fetal biometric characteristics. Umbilical artery abnormalities were associated with the composite adverse perinatal outcome, although this association should be interpreted in the context of delivery timing and clinical management. External validation and assessment of clinical utility are required before clinical implementation of the model.

## 1. Introduction

Fetal growth restriction (FGR) is a complex obstetric condition associated with increased perinatal morbidity and mortality and potential long-term health consequences. Although placental dysfunction is a major contributor, fetal size does not necessarily represent the degree of placental disease or an individual fetus’s growth potential. Small for gestational age (SGA) describes size relative to a reference distribution, whereas FGR refers to pathological failure to reach growth potential; some constitutionally small fetuses are healthy, and some growth-restricted fetuses remain above conventional biometric thresholds [1,2].

Diagnostic frameworks have evolved from isolated fetal size criteria toward combined assessment of biometry, growth trajectory, and fetoplacental circulation. The Delphi consensus specified different criteria for early- and late-onset FGR, incorporating severe smallness, abnormal Doppler measurements, and decreasing growth velocity [3]. The International Society of Ultrasound in Obstetrics and Gynecology (ISUOG) similarly distinguishes SGA from FGR and emphasizes gestational age-specific surveillance [4]. International definitions are not uniform: the Society for Maternal-Fetal Medicine (SMFM) defines FGR using estimated fetal weight (EFW) or abdominal circumference (AC) below the 10th percentile and highlights umbilical artery (UA) Doppler surveillance [5]. These distinctions make it important to separate cohort eligibility, diagnostic phenotype, and prognosis.

Gestational age at presentation also influences disease phenotype. Early-onset FGR is more commonly associated with substantial placental resistance and abnormal UA Doppler, whereas late-onset disease may arise despite preserved UA flow. Reduced cerebroplacental ratio (CPR) may provide information about cerebral redistribution in selected cases, although the incremental value of CPR beyond UA Doppler is debated [4,6]. Pooling early and late cases without addressing timing can obscure these relationships.

Doppler velocimetry provides functional information complementary to fetal biometry. In the Prospective Observational Trial to Optimize Pediatric Health in Intrauterine Growth Restriction (PORTO) study, abnormal UA Doppler was associated with adverse outcomes among fetuses with EFW below the 10th percentile [7]. A subsequent PORTO analysis linked cerebral redistribution with adverse outcomes [8]. Conversely, an individual participant data meta-analysis found little improvement when CPR was added to the umbilical artery pulsatility index (UA-PI) for prediction of a composite adverse perinatal outcome [9]. Hence, the apparent performance of an isolated marker should be distinguished from its incremental value within a multivariable model.

Doppler abnormalities can themselves trigger intensified surveillance or earlier delivery, complicating the interpretation of neonatal intensive care unit (NICU) admission and respiratory morbidity. The Trial of Randomized Umbilical and Fetal Flow in Europe (TRUFFLE) highlighted the balance between progression of very preterm FGR and morbidity due to premature delivery [10]. An antenatal risk model must therefore be distinguished from a conditional analysis that includes the subsequently observed gestational age at delivery.

The present study examined the association of antenatal Doppler findings with adverse perinatal outcomes among singleton pregnancies with suspected fetal smallness, operationally defined by EFW or AC below the 10th percentile. The primary objective was to compare a maternal biometric prediction model with a Doppler-integrated model, with secondary exploration of timing-related associations and cohort restrictions.

## 2. Materials and Methods

### 2.1. Study Design and Setting

This single-center retrospective observational cohort included pregnancies assessed for suspected or diagnosed FGR at Şanlıurfa Training and Research Hospital, Türkiye, from January 2024 to May 2026. Data were obtained from electronic medical records, the obstetric ultrasound archive, delivery records, and NICU documentation. The observational reporting approach followed the Strengthening the Reporting of Observational Studies in Epidemiology (STROBE) statement [11].

### 2.2. Study Population and Selection

The study cohort comprised 606 singleton pregnancies. For the principal analysis, a biometric inclusion rule of EFW or AC below the 10th percentile at the index examination retained 429 pregnancies. Of the remaining 177 pregnancies, 143 met none of the three screening criteria, and 34 had Doppler abnormality without biometric smallness; the latter were considered separately in sensitivity analyses because selected Doppler findings can satisfy early FGR criteria even without small biometry. Exclusion criteria were multiple gestations, major structural anomalies, suspected chromosomal abnormalities, growth-relevant intrauterine infections, inadequate outcome records, and unreliable gestational dating. The principal analytic cohort was defined by fetal smallness at the index examination rather than by confirmed pathological FGR. It could therefore include both constitutionally small fetuses and fetuses with pathological growth restriction. Throughout this manuscript, “pregnancies with suspected fetal smallness” refers to this biometric cohort and does not imply that all included pregnancies met Delphi or ISUOG diagnostic criteria for FGR [3,4,5].

Source-record review classified the 143 pregnancies meeting neither the biometric nor Doppler screening criteria as referrals for suspected smallness not confirmed at specialist assessment (104/143, 72.7%), maternal or placental-risk surveillance without qualifying fetal findings (24/143, 16.8%), or previous FGR/preeclampsia/stillbirth prompting targeted growth assessment (15/143, 10.5%). These pregnancies were retained only in the full-cohort sensitivity analysis and were excluded from the principal and expanded suspected-growth populations. When more than one examination was available, the index examination was defined as the earliest complete examination meeting the applicable screening rule; for the 143 nonqualifying referrals, the first complete specialist growth-and-Doppler assessment during the study period served as the index examination. This rule was applied before outcome ascertainment to preserve temporal precedence of all antenatal predictors.

### 2.3. Gestational Dating and Index Examination

Gestational age was determined by first-trimester crown–rump length where available and otherwise by a reliable last menstrual period supported by early ultrasound. The index examination was defined as the qualifying ultrasound supplying antenatal predictors; only information existing on or before that date was included in the baseline prognostic model. Index examination before versus at/after 32 + 0 weeks was used as an assessment-time stratification and was not treated as a definitive classification of early- or late-onset FGR.

### 2.4. Fetal Biometry and Reference Standards

Biparietal diameter, head circumference, abdominal circumference (AC), and femur length were measured using standard ultrasound planes. Estimated fetal weight (EFW) was calculated using the Hadlock IV formula, incorporating biparietal diameter, head circumference, abdominal circumference, and femur length [12]. Gestational age-specific EFW and AC percentiles were assigned according to the Hadlock reference curves [13]. An EFW or AC below the 10th percentile defined the principal analytic cohort, while values below the 3rd percentile indicated severe smallness. Continuous EFW and AC percentiles were retained in the prediction models rather than simultaneously entering nested binary thresholds. Ultrasound examinations were performed using a Samsung HS70 system (Samsung Medison Co., Seoul, Republic of Korea) equipped with a 2–6 MHz convex transducer.

### 2.5. Fetal Doppler Measurements

Umbilical artery pulsatility index (UA-PI) and end-diastolic flow, middle cerebral artery pulsatility index (MCA-PI), cerebroplacental ratio (CPR), and ductus venosus findings were recorded. CPR was calculated as MCA-PI divided by UA-PI. Gestational age-specific Doppler percentiles were assigned according to the Fetal Medicine Foundation reference ranges reported by Ciobanu et al. [14]. A UA-PI above the 95th percentile, an MCA-PI below the 5th percentile, and a CPR below the 5th percentile were considered abnormal. For regression analysis, UA status was categorized into three mutually exclusive groups: (i) normal UA-PI with preserved forward end-diastolic flow, (ii) UA-PI above the 95th percentile with preserved forward end-diastolic flow, and (iii) absent or reversed end-diastolic flow (AEDF/REDF), regardless of the UA-PI value. Normal UA status constituted the reference category. CPR and the composite variable “any abnormal Doppler finding” were not entered into the principal regression model alongside their constituent Doppler variables.

### 2.6. Diagnostic Phenotypes

Delphi and ISUOG frameworks differentiate early FGR (<32 weeks) from late FGR (≥32 weeks). For early FGR, AC/EFW < 3rd percentile or UA-AEDF qualifies individually, while AC/EFW < 10th percentile combined with UA-PI or uterine artery PI > 95th percentile provides an alternative route. For late FGR, AC/EFW < 3rd percentile is sufficient; otherwise, at least two of three domains are required: AC/EFW < 10th percentile, a crossing downward of more than two quartiles, or CPR < 5th percentile/UA-PI > 95th percentile [3,4]. Because the present analysis was anchored to the index examination, the 32-week subgroup analyses were interpreted as assessment-time strata rather than definitive onset phenotypes.

### 2.7. Maternal Characteristics and Obstetric Complications

Baseline candidate variables included age, obstetric history, smoking, preexisting hypertension and diabetes, and prior FGR or preeclampsia. Pregnancy complications and management variables were summarized separately. Preeclampsia was defined as new-onset hypertension after 20 weeks of gestation (systolic blood pressure ≥ 140 mmHg or diastolic blood pressure ≥ 90 mmHg on two occasions at least 4 h apart) accompanied by proteinuria, or, in the absence of proteinuria, by new-onset maternal organ dysfunction. Proteinuria was defined as ≥300 mg in a 24 h urine collection, a protein-to-creatinine ratio ≥ 0.3, or a urine dipstick reading of 2+ when quantitative testing was unavailable. Preeclampsia with severe features was defined by severe-range blood pressure (systolic ≥ 160 mmHg or diastolic ≥ 110 mmHg), thrombocytopenia, impaired liver function, renal insufficiency, pulmonary edema, or new-onset persistent cerebral or visual symptoms, consistent with the American College of Obstetricians and Gynecologists (ACOG) Practice Bulletin No. 222 [15]. Post-index events and treatment were excluded from the antenatal predictor sets.

### 2.8. Clinical Surveillance and Delivery Timing

During the study period, clinical surveillance and timing of delivery were managed in accordance with the contemporary literature and international ISUOG and SMFM guidance [4,5]. Management was individualized by the treating maternal-fetal medicine team according to gestational age, fetal biometric findings and growth trajectory, UA Doppler category, MCA-PI/CPR and ductus venosus findings when available, fetal surveillance results, and maternal condition. Abnormal UA Doppler findings prompted intensified surveillance and contributed to delivery planning; absent or reversed end-diastolic flow was considered a marker of severe placental insufficiency requiring close assessment and consideration of delivery in the context of gestational age and the overall maternal-fetal condition. Delivery decisions were not based on a single Doppler threshold alone, but incorporated fetal testing, evidence of deterioration, and maternal indications, including preeclampsia with severe features. Gestational age at delivery was a post-index variable and was therefore excluded from the antenatal baseline and Doppler-integrated prediction models; it was included only in a secondary sensitivity model to explore conditioning on delivery timing.

To characterize the observed management pathway, the interval from the index examination to delivery and the principal delivery indication were additionally summarized by Doppler category. Delivery indications were grouped a priori as fetal/placental (growth restriction, Doppler deterioration, or nonreassuring fetal surveillance), maternal (hypertensive disease or other maternal indication), spontaneous labor or preterm prelabor rupture of membranes (PPROM), or planned/other obstetric indication. These variables were treated as post-index descriptors rather than candidate antenatal predictors. The median index-to-delivery interval was 17 days (interquartile range [IQR], 8–30) overall, 18 days (IQR, 9–31) with normal Doppler, and 16 days (IQR, 7–29) with abnormal Doppler (Mann–Whitney *p* = 0.148). Delivery indications differed across Doppler strata (global *p* < 0.001), principally because fetal/placental indications were more frequent when Doppler was abnormal. These descriptive comparisons characterize management exposure but do not identify mediation or a direct causal effect of Doppler.

### 2.9. Primary and Secondary Outcomes

The primary outcome was a binary composite of at least one of the following: five-minute Apgar score < 7, umbilical artery pH < 7.10, NICU admission, documented neonatal respiratory support, neonatal death, or stillbirth. An affected pregnancy was counted once regardless of the number of components present. Secondary outcomes included gestational age at delivery, preterm delivery thresholds (<37, <34 and <32 weeks), birth weight, NICU admission, respiratory support, mechanical ventilation, hypoglycemia, sepsis, and death. For component-level reporting, stillbirth and neonatal death were summarized as the perinatal death component. Respiratory support was operationalized as documented neonatal respiratory assistance beyond room air, including supplemental oxygen, high flow nasal cannula, continuous or bilevel positive airway pressure, or invasive mechanical ventilation. An exploratory “hard” adverse-outcome sensitivity composite was defined as five-minute Apgar score < 7, umbilical artery pH < 7.10, or perinatal death; because of the small number of hard events, this sensitivity analysis was limited to crude group comparison rather than multivariable modeling.

### 2.10. Data-Quality Procedures

One record represented one pregnancy. The dataset was checked for unique identifiers, percentile consistency, gestational dating, temporal precedence of predictors, Doppler-category exclusivity, component-based outcome coding, completeness, and clinical plausibility. In the principal analytic cohort of 429 pregnancies, there were no missing values (0/429, 0%) for maternal age, gestational age at the index examination, EFW percentile, AC percentile, chronic hypertension, UA Doppler category, MCA-PI abnormality status, gestational age at delivery, or the primary composite outcome. All 429 pregnancies were therefore included in each multivariable model, with no exclusions due to missing model data. No imputation was required or performed. No missing values were present for the primary-outcome components or for variables summarized in the principal Tables 2–6; consequently, neither complete case exclusions nor imputation were required. Because no imputation was performed, fold-specific imputation and the associated risk of cross-validation information leakage were not applicable. Data were also complete for all predictors and the primary outcome in the full cohort and the expanded suspected-growth cohort; all 606 and 463 pregnancies, respectively, were included in the corresponding sensitivity models without imputation.

Completeness was verified against the source laboratory, delivery, and neonatal records rather than inferred from a default “normal” code. In the principal 429-pregnancy cohort, an arterial cord-blood gas result was documented for 429/429 deliveries; therefore, unavailable pH measurements were 0/429 and numeric values ≥ 7.10 were explicitly distinguished from the adverse pH component. Five-minute Apgar score, NICU admission, respiratory-support status, and perinatal survival were likewise source-verified for all 429 pregnancies. A second investigator cross-checked outcome-component coding against the neonatal record for all composite-positive pregnancies and a 10% random sample of composite-negative pregnancies; discrepancies were reconciled by return to the source record before analysis.

### 2.11. Descriptive and Inferential Analysis

Continuous variables were summarized using mean and standard deviation, and categorical variables as *n* (%). Welch’s *t*-test was used when Shapiro–Wilk testing did not reject normality in either group; otherwise, the Mann–Whitney U test was applied. Categorical variables were assessed with Pearson chi-square or Fisher exact test when expected 2 × 2 cell counts were below five. Tests were two-sided and nominal; secondary comparisons were exploratory and were not adjusted for multiplicity. Crude odds ratios with 95% confidence intervals summarized abnormal-versus-normal Doppler comparisons.

### 2.12. Discrimination and Multivariable Models

Receiver operating characteristic (ROC) analyses used preselected continuous score directions and clinical binary thresholds; apparent areas under the curve (AUCs) and 95% confidence intervals were calculated using 1000 stratified bootstrap resamples. The baseline logistic model included maternal age, index gestational age, EFW percentile, AC percentile, and chronic hypertension. The Doppler model added two mutually exclusive UA indicator variables and an indicator of MCA-PI below the 5th percentile. A third conditional sensitivity model also included gestational age at delivery; it is not an antenatal prediction model. Regression estimates were presented as odds ratios, 95% confidence intervals and nominal *p* values. The nested-model likelihood-ratio test compared baseline versus Doppler-integrated apparent fit. Model specification and reporting were informed by the Transparent Reporting of a multivariable prediction model for Individual Prognosis or Diagnosis and Artificial Intelligence (TRIPOD + AI) statement [16]. The final baseline specification was intentionally parsimonious and clinically structured to represent maternal baseline risk (maternal age and chronic hypertension), timing of assessment (index gestational age), and fetal size (EFW and AC percentiles); because this was a retrospective analysis, we do not claim protocol-registered predictor prespecification. Predictor inclusion was not justified by univariable significance alone. Potential collinearity between EFW and AC percentiles was assessed using their correlation and variance inflation factors (VIFs). Sensitivity models were also fitted with EFW percentile alone or AC percentile alone, retaining the same maternal covariates before addition of the Doppler terms.

Additional prespecified sensitivity models were constructed to address the reviewer-requested comparisons. Using exactly the same principal cohort and the same cross-validation folds, three Doppler specifications were compared against the maternal-biometric baseline: (i) UA category only, (ii) continuous CPR only, and (iii) the principal integrated specification containing mutually exclusive UA categories plus MCA-PI < 5th percentile. Component-specific logistic models for NICU admission and respiratory support used the same baseline covariates and Doppler terms as the primary model. A second conditional version of each component model additionally included gestational age at delivery solely to assess attenuation after conditioning on this post-index variable; these conditional estimates were not interpreted as antenatal prediction models.

An expanded-baseline sensitivity model added smoking, pregestational diabetes, previous FGR, and previous preeclampsia to the original maternal-biometric baseline. These variables were selected because they were available at the index assessment and represented clinically plausible antenatal risk domains without incorporating post-index management or pregnancy complications. The corresponding expanded Doppler model added the same UA-category and MCA-PI terms used in the principal analysis.

For robustness of internal validation, stratified 10-fold cross-validation was repeated 50 times using different reproducible fold partitions, with the same partition applied to every candidate model within each repetition. The median and 5th–95th percentile range of AUC, Brier score, calibration intercept, calibration slope, and paired model differences were summarized across repetitions. In addition, 1000 bootstrap samples were used for a refit-aware internal-validation analysis in which the complete model-fitting procedure was repeated within each bootstrap sample and performance was evaluated against the original sample to estimate optimism-corrected discrimination and calibration. Statistical analyses were performed using IBM SPSS Statistics (version 30.0; IBM Corp., Armonk, NY, USA) for descriptive analyses and R (version 4.4.2; R Foundation for Statistical Computing, Vienna, Austria) for resampling, regression validation, and calibration procedures.

### 2.13. Internal Validation and Calibration

Stratified 10-fold cross-validation generated an out-of-fold predicted probability for each record, with predictor preprocessing fitted within training folds. Performance measures included out-of-fold AUC, Brier score, calibration intercept, and calibration slope. Apparent and out-of-fold AUCs were reported separately. Internal resampling was considered internal validation and not external validation. The 32-week and alternative cohort-restriction analyses were treated as overlapping exploratory comparisons rather than independent samples. Uncertainty in incremental internally validated performance was quantified from the paired out-of-fold predictions using 3000 nonparametric paired bootstrap resamples; ΔAUC was defined as Doppler-integrated minus baseline AUC, and Brier improvement as baseline minus Doppler-integrated Brier score. The same baseline and Doppler-integrated model specifications were additionally refit and internally validated within the full 606-pregnancy cohort and the 463 pregnancy expanded suspected-growth cohort. Calibration was displayed graphically using deciles of out-of-fold predicted risk together with the logistic calibration relationship defined by the out-of-fold calibration intercept and slope. These analyses follow the performance-reporting principles of TRIPOD + AI [16]. Baseline and Doppler-integrated models were evaluated using identical cross-validation folds. In each bootstrap resample, the same pregnancy indices were sampled jointly for the outcome and both sets of out-of-fold predictions, preserving the paired comparison. Confidence intervals were defined by the 2.5th and 97.5th percentiles of the bootstrap distribution.

The paired bootstrap confidence intervals from the original analysis condition on the originally generated out-of-fold predictions and therefore quantify sampling uncertainty for those paired predictions. The repeated 50 × 10-fold analysis additionally assessed sensitivity to fold assignment, whereas the refit-aware bootstrap propagated uncertainty from complete model refitting. These complementary procedures were interpreted as internal validation only. No model-selection step was performed within validation loops, and all candidate models were evaluated on identical partitions. Clinical utility was not inferred from discrimination or calibration metrics alone.

### 2.14. Sample Size and Ethical Considerations

Because this study used a retrospective convenience cohort, no a priori sample-size calculation was performed. The complexity of the multivariable models was determined with consideration of the observed number of outcome events and the limited size of the absent/reversed end-diastolic flow (AEDF/REDF) subgroup.

For reproducibility, the principal baseline and Doppler-integrated model equations, including regression coefficients and intercepts, are provided in Supplementary Tables S1–S5; all performance comparisons use the same analytic cohort and identical outcome definition.

The study was approved by the Clinical Research Ethics Committee of Health Sciences University Gazi Yaşargil Training and Research Hospital (approval no. 298; 5 June 2026). The requirement for written informed consent was waived by the Ethics Committee because of the retrospective design and the use of anonymized, routinely collected clinical data. The study was conducted in accordance with the principles of the Declaration of Helsinki.

## 3. Results

### 3.1. Cohort and Endpoint Characteristics

**Figure 1:**
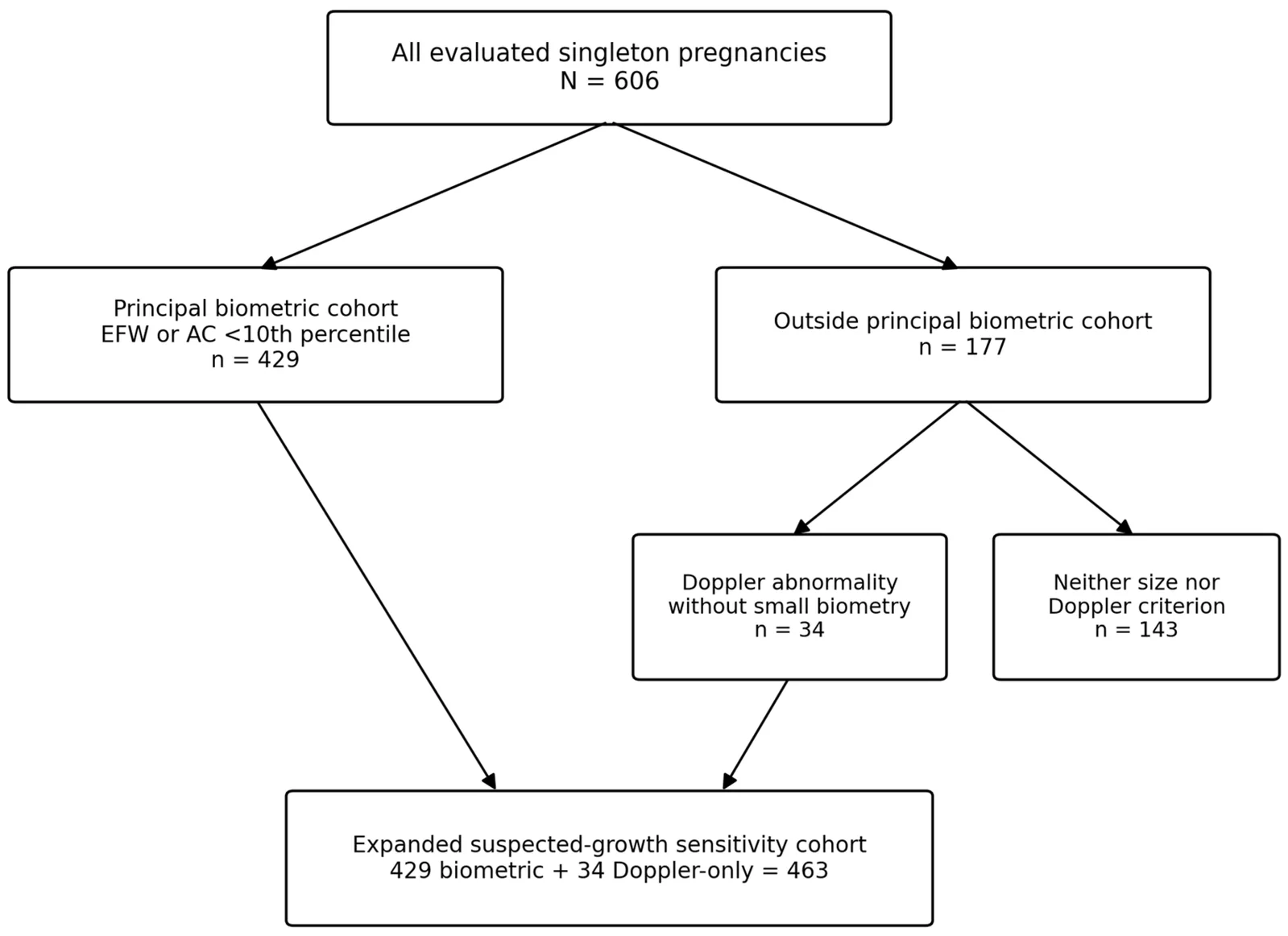
Cohort derivation and sensitivity populations. Abbreviations: EFW, estimated fetal weight; AC, abdominal circumference.

The dataset comprised 606 pregnancies. Restriction to EFW or AC below the 10th percentile retained 429 (70.8%) in the principal analytic cohort. Among 177 pregnancies outside this cohort, 143 met none of the original screening criteria, and 34 had isolated Doppler abnormalities without biometric smallness. The composite adverse perinatal outcome was present in 111 of 429 pregnancies (25.9%) and in 151 of 606 pregnancies (24.9%) in the full cohort (Table 1). The derivation of the full, principal, and expanded sensitivity populations is summarized in Figure 1.

### 3.2. Maternal and Biometric Findings

In the 429-pregnancy principal cohort, mean maternal age was 28.43 ± 5.61 years and index gestational age was 33.07 ± 3.10 weeks. Pregnancies with the composite adverse outcome had earlier index assessments (31.79 ± 3.06 vs. 33.52 ± 2.99 weeks; *p* < 0.001), while maternal age and most medical/obstetric histories did not differ significantly (Table 2). EFW (1551.77 ± 640.36 vs. 2067.25 ± 884.78 g; *p* < 0.001), AC (256.33 ± 34.21 vs. 272.00 ± 33.04 mm; *p* < 0.001), and EFW percentile (7.68 ± 6.44 vs. 9.53 ± 7.12; *p* = 0.022) were lower in pregnancies with the composite outcome. AC percentile did not differ significantly (*p* = 0.691), nor did the binary thresholds below the 3rd percentile (Table 3). EFW and AC percentiles showed only modest correlation within the range-restricted principal cohort (Pearson r = −0.264), and VIFs were 1.08 for each variable, arguing against problematic multicollinearity. In single-biometry sensitivity models, out-of-fold AUC/Brier values were 0.647/0.182 with EFW alone and 0.632/0.184 with AC alone; after Doppler addition, the corresponding values were 0.692/0.174 and 0.690/0.174, respectively.

In the unrestricted 606-pregnancy cohort, EFW and AC percentiles were positively correlated (Pearson r = 0.610; 95% confidence interval [CI], 0.557–0.658; *p* < 0.001). The correlation reversed within the principal cohort selected by EFW or AC < 10th percentile (r = −0.264), supporting selection-related range restriction/collider conditioning rather than an intrinsically inverse biological relation between the two biometric measures.

### 3.3. Doppler and Maternal Events

Any abnormal fetal Doppler was present in 206/429 pregnancies (48.0%), including 85/111 (76.6%) with and 121/318 (38.1%) without the composite outcome (*p* < 0.001). The adverse-outcome group had higher UA-PI (1.26 ± 0.22 vs. 1.14 ± 0.19) and lower CPR (1.24 ± 0.28 vs. 1.47 ± 0.33), both *p* < 0.001. UA status comprised 266 normal-flow/normal-PI, 144 elevated-PI/forward-flow, and 19 AEDF/REDF pregnancies (Table 4). Preeclampsia and severe features did not differ significantly between outcome groups, whereas preterm labor was more frequent among pregnancies with the composite outcome (27.9% vs. 14.2%; *p* = 0.001); maternal complications and treatment indicators are presented in Table 5.

### 3.4. Delivery and Neonatal Outcomes

The Doppler-abnormal group delivered earlier than the normal group (35.06 ± 2.60 vs. 36.50 ± 2.62 weeks; *p* < 0.001) and had lower birth weight (2100.56 ± 691.87 vs. 2474.72 ± 670.64 g; *p* < 0.001). NICU admission occurred in 80/206 (38.8%) versus 25/223 (11.2%), and respiratory support in 51/206 (24.8%) versus 18/223 (8.1%). The composite adverse perinatal outcome was present in 85/206 (41.3%) versus 26/223 (11.7%; *p* < 0.001) (Table 6). Component frequencies in the full principal cohort were 4/429 (0.9%) for five-minute Apgar < 7, 5/429 (1.2%) for umbilical artery pH < 7.10, 105/429 (24.5%) for NICU admission, 69/429 (16.1%) for respiratory support, and 2/429 (0.5%) for perinatal death. The hard sensitivity composite occurred in 11/429 pregnancies (2.6%): 8/206 (3.9%) with abnormal Doppler and 3/223 (1.3%) with normal Doppler (odds ratio [OR], 2.96; 95% CI, 0.78–11.32; Fisher exact *p* = 0.128). The low event count precluded stable multivariable modeling of this hard endpoint.

Component overlap showed that 66 pregnancies had both NICU admission and respiratory support, 39 had NICU admission without respiratory support, three had respiratory support without NICU admission, and three met the primary composite solely through other components; these mutually exclusive groups sum to all 111 composite events. Thus, NICU admission contributed to 105/111 (94.6%) composite-positive pregnancies and respiratory support to 69/111 (62.2%), confirming that the primary endpoint predominantly represented neonatal care requirements rather than uniformly severe fetal compromise.

### 3.5. Assessment-Time Strata and Cohort Restrictions

In component-specific adjusted models, elevated UA-PI with forward flow remained associated with NICU admission (adjusted odds ratio [aOR], 2.54; 95% CI, 1.51–4.27; *p* < 0.001) and respiratory support (aOR 2.20, 95% CI 1.23–3.94; *p* = 0.008), while AEDF/REDF showed larger but imprecise associations with NICU admission (aOR 6.12, 95% CI 2.20–17.02; *p* = 0.001) and respiratory support (aOR 5.43, 95% CI 1.82–16.18; *p* = 0.002). Abnormal MCA-PI was not independently associated with either component. After conditional adjustment for gestational age at delivery, the UA estimates were attenuated (NICU: elevated UA-PI aOR 1.68, 95% CI 0.95–2.96; AEDF/REDF aOR 4.12, 95% CI 1.36–12.48; respiratory support: elevated UA-PI aOR 1.54, 95% CI 0.80–2.95; AEDF/REDF aOR 3.71, 95% CI 1.13–12.19), consistent with delivery timing lying on or being affected by the management pathway. These conditional models are descriptive and do not estimate direct causal effects.

Among 132 pregnancies assessed before 32 weeks, adverse outcomes occurred in 37/71 (52.1%) with abnormal Doppler and 12/61 (19.7%) with normal Doppler. Among 297 pregnancies assessed at or after 32 weeks, the corresponding counts were 48/135 (35.6%) and 14/162 (8.6%). These categories represent index-examination timing rather than definitive early- or late-onset Delphi FGR (Table 7). The crude association between abnormal Doppler and the composite outcome was OR 5.32 (95% CI 3.25–8.72) in the 429-pregnancy principal cohort, OR 4.59 (95% CI 3.10–6.80) in all 606 pregnancies, and OR 5.51 (95% CI 3.40–8.92) after exclusion of the 143 pregnancies meeting neither screening criterion (Table 8). When the identical models were evaluated in broader sensitivity populations, the full 606-pregnancy cohort had out-of-fold AUCs of 0.665 (baseline) and 0.709 (Doppler-integrated), with Brier scores of 0.177 and 0.168. In the expanded 463 pregnancy-suspected growth cohort, the corresponding AUCs were 0.644 and 0.698, and Brier scores were 0.190 and 0.179. Thus, the direction of incremental Doppler performance persisted outside the range-restricted principal cohort.

### 3.6. Individual-Marker Discrimination

Using identical folds in the principal cohort, out-of-fold AUC/Brier values were 0.642/0.183 for baseline alone, 0.679/0.177 for baseline + UA categories, 0.684/0.176 for baseline + continuous CPR, and 0.690/0.174 for the principal UA-category + abnormal-MCA specification (Table 9). Relative to baseline, paired ΔAUCs were +0.037 (95% bootstrap CI 0.002–0.074), +0.042 (0.004–0.081), and +0.049 (0.005–0.094), respectively. Direct comparisons were small and imprecise: integrated versus UA-only ΔAUC +0.011 (95% CI −0.012 to 0.034), integrated versus CPR ΔAUC +0.006 (−0.014 to 0.026), and CPR versus UA-only ΔAUC +0.005 (−0.019 to 0.029). Corresponding Brier-score improvements over baseline were 0.006 (95% CI −0.0008 to 0.0131), 0.007 (−0.0005 to 0.0144), and 0.009 (−0.0004 to 0.0179). Thus, all Doppler formulations improved point estimates relative to baseline, but no candidate specification demonstrated clear superiority over another.

**Table 9:** Apparent discriminatory performance of individual biometric and Doppler markers.

Index	Apparent AUC (95% Bootstrap CI)	Sensitivity %	Specificity %	Measure Type
EFW < 3rd percentile	0.536 (0.486–0.588)	32.4	74.8	Binary threshold
AC < 3rd percentile	0.495 (0.444–0.549)	33.3	65.7	Binary threshold
UA-PI > 95th percentile	0.645 (0.594–0.695)	59.5	69.5	Binary threshold
AEDF/REDF	0.537 (0.508–0.566)	9.9	97.5	Binary threshold
Abnormal MCA-PI	0.516 (0.480–0.558)	17.1	86.2	Binary threshold
Abnormal CPR	0.569 (0.520–0.620)	32.4	81.4	Binary threshold
Any abnormal Doppler	0.693 (0.646–0.744)	76.6	61.9	Binary threshold
EFW percentile (lower = risk)	0.573 (0.513–0.633)	—	—	Continuous measurement
AC percentile (lower = risk)	0.487 (0.427–0.552)	—	—	Continuous measurement
UA-PI (higher = risk)	0.657 (0.595–0.715)	—	—	Continuous measurement
CPR (lower = risk)	0.704 (0.652–0.757)	—	—	Continuous measurement
Paired AUC difference: UA-PI minus EFW percentile	0.084 (0.007–0.164)	—	—	Exploratory paired bootstrap

Note: AUC, area under the receiver operating characteristic curve; CI, confidence interval; EFW, estimated fetal weight; AC, abdominal circumference; UA-PI, umbilical artery pulsatility index; MCA-PI, middle cerebral artery pulsatility index; CPR, cerebroplacental ratio; AEDF, absent end-diastolic flow; REDF, reversed end-diastolic flow. For continuous markers, the risk direction is indicated in parentheses. These estimates represent apparent individual-marker performance in the analytic cohort. Bootstrap confidence intervals quantify uncertainty around the apparent estimates and do not constitute out-of-fold validation. These AUCs should not be directly compared with the cross-validated multivariable-model AUCs in Table 10.

**Table 10:** Multivariable models and internal performance.

Covariate	Baseline aOR (95% CI); *p*	Doppler aOR (95% CI); *p*	GA-Delivery Sensitivity aOR (95% CI); *p*
Maternal age (per year)	1.02 (0.97–1.06); 0.466	1.02 (0.98–1.06); 0.360	1.02 (0.97–1.06); 0.465
Index gestational age (per week)	0.84 (0.78–0.90); <0.001	0.85 (0.78–0.91); <0.001	1.20 (1.02–1.41); 0.027
EFW percentile (per centile)	0.96 (0.93–1.00); 0.038	0.98 (0.95–1.02); 0.386	0.98 (0.95–1.02); 0.373
AC percentile (per centile)	1.00 (0.96–1.03); 0.830	1.01 (0.97–1.05); 0.639	1.02 (0.98–1.06); 0.346
Chronic hypertension	0.84 (0.34–2.06); 0.704	0.95 (0.38–2.37); 0.920	0.96 (0.38–2.45); 0.933
UA: high PI with forward flow vs. normal	—	2.61 (1.58–4.32); <0.001	1.96 (1.15–3.32); 0.013
UA: AEDF/REDF vs. normal	—	6.60 (2.40–18.18); <0.001	5.51 (1.94–15.62); 0.001
MCA-PI < 5th percentile	—	1.14 (0.60–2.14); 0.690	1.14 (0.60–2.17); 0.691
GA at delivery (secondary, per week)	—	—	0.62 (0.51–0.75); <0.001
Sample size	429	429	429
Outcome events	111	111	111
Apparent AUC	0.669	0.720	0.772
Apparent Brier score	0.179	0.168	0.157
Nested LR test vs. baseline	Reference	χ^2^ = 23.52; *p* < 0.001	Not nested for antenatal use
10-fold out-of-fold AUC	0.642	0.690	0.748
10-fold out-of-fold Brier	0.183	0.174	0.165
Paired out-of-fold ΔAUC vs. baseline	Reference	+0.049 (95% CI 0.005–0.094)	—
Paired out-of-fold Brier improvement vs. baseline	Reference	+0.009 (95% CI −0.0004 to 0.0179)	—
Out-of-fold calibration intercept	−0.169	−0.147	−0.119
Out-of-fold calibration slope	0.829	0.841	0.866

Note: Values are adjusted odds ratios (95% confidence intervals) with *p* values unless otherwise specified. aOR, adjusted odds ratio; CI, confidence interval; GA, gestational age; EFW, estimated fetal weight; AC, abdominal circumference; UA, umbilical artery; PI, pulsatility index; MCA, middle cerebral artery; AEDF, absent end-diastolic flow; REDF, reversed end-diastolic flow; AUC, area under the receiver operating characteristic curve; LR, likelihood ratio. Out-of-fold estimates were obtained using stratified 10-fold cross-validation. Gestational age at delivery was included only in the secondary sensitivity model and not in the antenatal prediction models. Paired uncertainty intervals for the validated performance differences were obtained from 3000 paired bootstrap resamples of the out-of-fold predictions; positive ΔAUC and positive Brier improvement favor the Doppler-integrated model.

### 3.7. Multivariable Models and Internal Performance

Across 50 repetitions of stratified 10-fold cross-validation, median out-of-fold AUCs were 0.643 for baseline and 0.689 for the integrated model; the median paired ΔAUC was 0.046 (5th–95th percentile across repetitions, 0.031–0.061). Median Brier scores were 0.183 and 0.174, respectively, with a median improvement of 0.009 (5th–95th percentile, 0.004–0.014). Median calibration intercept/slope values were −0.166/0.832 for baseline and −0.144/0.846 for the integrated model; integrated-model slopes across repetitions had a 5th–95th percentile range of 0.786–0.914. In the 1000-sample refit-aware bootstrap, optimism-corrected AUCs were 0.645 (95% CI 0.589–0.697) for baseline and 0.687 (0.635–0.736) for the integrated model, with an optimism-corrected paired ΔAUC of 0.042 (0.007–0.078). Optimism-corrected Brier scores were 0.182 and 0.175, respectively (improvement 0.007, 95% CI 0.001–0.014). These analyses indicate that the direction of incremental discrimination was stable, while the calibration slope remained below 1 and therefore continued to suggest modest overfitting.

**Figure 2:**
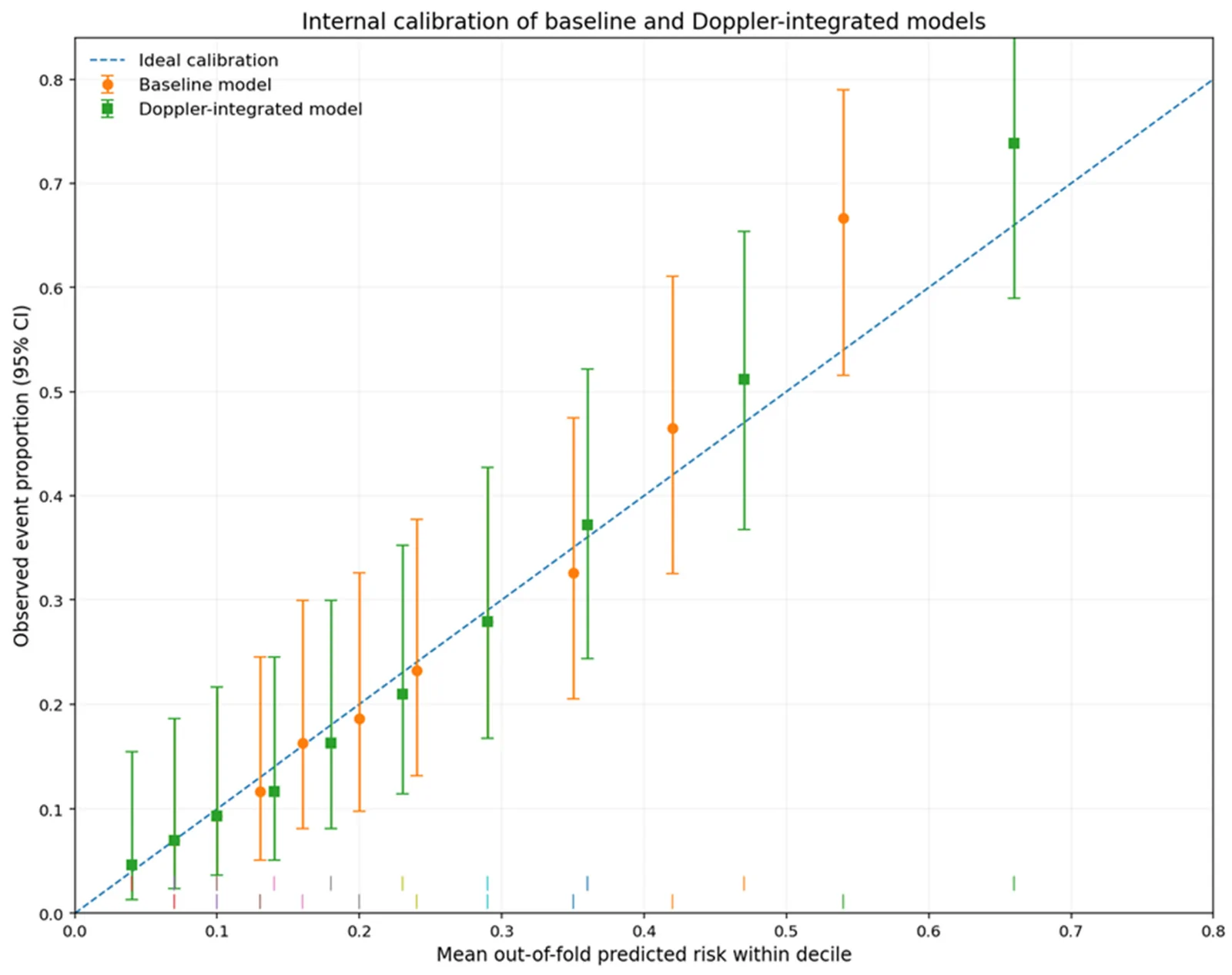
Internal calibration of the baseline and Doppler-integrated models using out-of-fold predictions. Points represent observed event proportions within deciles of predicted risk; vertical bars show 95% Wilson confidence intervals. Short tick marks along the lower margin depict the distribution of decile mean predicted risks. The dashed diagonal indicates ideal calibration. Abbreviation: CI, confidence interval.

All three multivariable models included the same 429 pregnancies and 111 outcome events, with complete data for all included predictors and the primary outcome. The baseline maternal–biometric model had apparent and out-of-fold AUCs of 0.669 and 0.642. The Doppler-integrated model yielded 0.720 and 0.690, respectively; corresponding out-of-fold Brier scores were 0.183 and 0.174. The nested likelihood-ratio test was χ^2^ = 23.52 (*p* < 0.001). Relative to normal UA, elevated PI with forward flow had an adjusted OR of 2.61 (95% CI 1.58–4.32), and AEDF/REDF had an adjusted OR of 6.60 (95% CI 2.40–18.18). The Doppler model had an out-of-fold calibration intercept of −0.147 and slope of 0.841. Adding gestational age at delivery yielded an out-of-fold AUC of 0.748; because delivery timing is post-index, this model was interpreted only as a sensitivity analysis and not as an antenatal prediction model (Table 10). The paired internally validated AUC increment was 0.049 (95% bootstrap CI 0.005–0.094). The paired Brier-score improvement was 0.009 (95% bootstrap CI −0.0004 to 0.0179), indicating that improvement in mean squared prediction error was small and its interval included essentially no change. The likelihood-ratio result therefore remains an assessment of apparent nested-model fit rather than a substitute for validated performance comparison. Figure 2 displays the out-of-fold calibration of the baseline and Doppler-integrated models.

The expanded antenatal baseline included maternal age, index gestational age, EFW percentile, AC percentile, chronic hypertension, smoking, pregestational diabetes, previous FGR, and previous preeclampsia. Its out-of-fold AUC/Brier score was 0.656/0.181, compared with 0.695/0.173 after addition of UA categories and abnormal MCA-PI; the paired ΔAUC was +0.039 (95% CI 0.003–0.078) and Brier improvement was 0.008 (95% CI −0.001 to 0.016). Out-of-fold calibration slopes were 0.814 and 0.829, respectively. The direction and magnitude of the Doppler increment were therefore similar to the primary parsimonious comparison, suggesting that the principal finding was not solely attributable to omission of these additional antenatal maternal-risk variables.

## 4. Discussion

### 4.1. Principal Interpretation

This study examined whether Doppler information complements fetal biometry among pregnancies selected for suspected fetal smallness. Abnormal UA flow patterns were associated with the composite adverse perinatal outcome, and adding mutually exclusive UA categories and abnormal MCA-PI to the maternal–biometric model improved discrimination modestly. These findings support a structured risk-assessment approach that distinguishes cohort eligibility from prognosis and separates antenatal information from downstream delivery timing.

### 4.2. Fetal Size Versus Pathological Growth Restriction

EFW and AC are central screening measures but do not alone quantify placental function; SGA and FGR have different conceptual meanings [1,3,4]. Molina et al. found that Delphi criteria were associated with adverse neonatal outcomes in a referred cohort, yet both Delphi and an EFW < 10th-percentile definition had limited discrimination for a morbidity composite [17]. The relatively low discriminatory performance of EFW and AC should be interpreted in the context of cohort selection based on these same biometric measures. Restriction to pregnancies with EFW or AC below the 10th percentile limits the distribution of biometric values and may attenuate their apparent prognostic contribution. The findings therefore describe incremental Doppler information within a population already selected for fetal smallness and should not be interpreted as demonstrating general superiority of Doppler over biometry in an unselected population. Model performance in broader populations may differ. This interpretation is consistent with a contemporary evidence-based approach that treats suspected FGR as an integrated phenotype incorporating fetal size, Doppler findings, gestational context, and longitudinal information rather than a single biometric cutoff [18]. The broader-cohort sensitivity models also support the role of range restriction: baseline discrimination was 0.665 in all 606 pregnancies and 0.644 in the expanded 463-pregnancy cohort, while the Doppler-integrated AUCs were 0.709 and 0.698, respectively. Because these populations overlap, these estimates are sensitivity analyses rather than independent validation.

### 4.3. Umbilical Artery Findings

The negative EFW–AC percentile correlation within the principal cohort (r = −0.264) contrasts with the positive association observed in all 606 pregnancies (r = 0.610, 95% CI 0.557–0.658). This pattern is compatible with conditioning on the union of two low-percentile criteria: once inclusion can be triggered by either a low EFW percentile or a low AC percentile, range restriction and collider-type selection can induce an inverse within-selected correlation even when the underlying measurements are positively related. The finding therefore reflects cohort construction rather than evidence that EFW and AC are biologically discordant.

Raised UA resistance and AEDF/REDF provide hemodynamic information beyond a single size measurement, consistent with placental vascular dysfunction [2,4]. PORTO found adverse-outcome associations with abnormal UA Doppler among fetuses defined by EFW < 10th percentile [7]. Our findings showed the same direction of association, with progressively higher adjusted odds across elevated UA-PI with forward flow and AEDF/REDF. Only 19 pregnancies had AEDF/REDF, of which 11 met the composite outcome. The adjusted odds ratio of 6.60 had a wide 95% confidence interval of 2.40–18.18, indicating substantial uncertainty regarding the magnitude of the association. Although the estimate supports an association with higher odds of the composite outcome, its numerical magnitude should not be treated as a precise risk estimate for individual counseling or management decisions. Confirmation in larger cohorts with more severe UA flow abnormalities is required.

### 4.4. CPR and Incremental Prediction

The matched-fold candidate-model comparisons yielded out-of-fold AUCs of 0.679 for baseline + UA categories, 0.684 for baseline + CPR, and 0.690 for baseline + UA categories plus abnormal MCA-PI. Although the integrated model had the highest point estimate, its paired AUC difference versus CPR was only 0.006 (95% CI −0.014 to 0.026), and versus UA-only, it was 0.011 (−0.012 to 0.034). These results are consistent with the individual-participant-data meta-analysis showing limited incremental improvement from CPR over UA-PI [9]: CPR may carry clinically relevant redistribution information without necessarily producing a large additional gain when maternal, biometric, and placental-resistance information are already represented. The principal model should therefore be interpreted as one clinically structured specification rather than as a proven superior Doppler formulation.

CPR had a higher apparent individual AUC than UA-PI in the present cohort (0.704 versus 0.657), but isolated-marker performance should be interpreted separately from cross-validated multivariable performance. In the matched-fold comparison, the baseline + continuous CPR model achieved an out-of-fold AUC of 0.684, compared with 0.679 for baseline + UA categories and 0.690 for the integrated UA-category + abnormal-MCA model; the direct paired differences were small, and their confidence intervals included zero. Because CPR is calculated from MCA-PI and UA-PI, it summarizes cerebral redistribution but does not explicitly preserve the distinction between preserved, absent, and reversed UA end-diastolic flow represented by the categorical UA predictor. PORTO linked cerebral redistribution with adverse outcomes [8], whereas the individual-participant meta-analysis of Vollgraff Heidweiller-Schreurs et al. found negligible AUC gain when CPR was added to UA-PI [9]. Accordingly, the present analyses support CPR as a clinically plausible alternative Doppler specification but do not demonstrate superiority of either a CPR-based or the integrated Doppler formulation.

The isolated CPR AUC of 0.704 remains an apparent univariable estimate and should not be compared directly with cross-validated multivariable AUCs. The same-fold multivariable comparison is more informative: CPR and the integrated UA/MCA specification both improved discrimination relative to the maternal-biometric baseline, but their direct difference was small and compatible with no meaningful separation. This distinction is important because CPR mathematically combines MCA-PI and UA-PI, whereas the categorical UA specification also preserves clinically important absent/reversed end-diastolic-flow information emphasized in FGR surveillance guidance [4,5].

### 4.5. Generalizability of Prognostic Performance

Across 50 repeated stratified cross-validation partitions, the incremental AUC remained positive (median 0.046; 5th–95th percentile 0.031–0.061), and the refit-aware bootstrap produced an optimism-corrected ΔAUC of 0.042 (95% CI 0.007–0.078). This concordance suggests that the observed improvement was not dependent on a single fold assignment. Nevertheless, the integrated-model calibration slope remained below 1 (median 0.846 across repeated cross-validation), indicating residual overfitting. In accordance with TRIPOD + AI principles [16], these results support internal robustness but not transportability or clinical utility; external validation in an independent cohort remains necessary.

Lopian et al. studied 14,161 low-risk singleton pregnancies at routine 35–37-week ultrasound; their focus on prediction of a small neonate differs from prediction of morbidity in an already-small cohort [19]. Differences in target population and endpoint prevent direct AUC comparison. Peng et al. systematically reviewed FGR prognostic models and found that multipredictor approaches may improve performance, but internal and external validation are often inadequate [20]. In our study, incorporation of Doppler variables increased the out-of-fold AUC from 0.642 to 0.690, an absolute improvement of 0.049. This represents a modest gain in discrimination, and the resulting model performance remains insufficient to establish readiness for clinical implementation. The out-of-fold calibration slope of 0.841 also suggests some overfitting. Although the nested likelihood-ratio test demonstrated improved apparent model fit, it does not establish clinical utility or the statistical significance of the out-of-fold AUC difference. External validation and assessment of clinical utility are required before the model can be considered for clinical use. The paired uncertainty analysis further qualifies this finding: the out-of-fold ΔAUC was 0.049 (95% CI 0.005–0.094), whereas the Brier-score improvement was 0.009 with a 95% CI spanning approximately zero. Accordingly, the data support modest additional prognostic information, not a claim of established clinical benefit.

The baseline covariates were chosen to represent maternal predisposition, assessment timing, and fetal size while limiting model complexity relative to 111 outcome events. The expanded-baseline sensitivity model additionally incorporated smoking, pregestational diabetes, previous FGR, and previous preeclampsia. Its baseline AUC increased from 0.642 to 0.656, while the corresponding Doppler-integrated AUC was 0.695; the incremental gain remained 0.039. This supports the robustness of the direction of effect to a broader clinically justified maternal-risk specification, while the modest calibration slopes and the sample-size considerations emphasized by Riley et al. [21] argue against unnecessary model expansion or claims of definitive clinical readiness.

### 4.6. Delivery Timing and Causal Interpretation

The component-overlap analysis identified 66 pregnancies with both NICU admission and respiratory support, underscoring that these outcomes are not independent events. This further supports interpreting the primary composite principally as a marker of neonatal care requirements under the observed surveillance and delivery policy, rather than as a direct measure of severe fetal injury.

Pregnancies with abnormal Doppler findings were delivered earlier and experienced more NICU admission and respiratory support. NICU admission was recorded in 105 pregnancies and respiratory support in 69, compared with 111 pregnancies meeting the composite outcome; these components could overlap within the same pregnancy. The composite therefore largely reflects neonatal care requirements and should not be interpreted as a uniform measure of severe fetal compromise. NICU admission and respiratory support may be influenced by fetal condition, gestational age at delivery, and clinical management. Because Doppler abnormalities prompted intensified surveillance and contributed to delivery planning, the observed associations may reflect both underlying fetal compromise and morbidity associated with earlier delivery. The TRUFFLE randomized trial and its cohort report illustrate why both fetal deterioration and morbidity related to premature delivery must be considered [10,22]. The magnitude of the association between abnormal Doppler findings and the composite outcome should therefore be interpreted cautiously. Gestational age at delivery is a post-index variable potentially influenced by Doppler findings and management; including it in a sensitivity model does not isolate a causal direct effect or fully account for management-related influences. The component analysis reinforces this interpretation: low five-minute Apgar, marked cord acidemia, and perinatal death were uncommon, whereas NICU admission and respiratory support contributed substantially more events. In the exploratory hard-outcome composite (Apgar < 7, pH < 7.10, or perinatal death), the association with abnormal Doppler was directionally similar but imprecise (OR 2.96, 95% CI 0.78–11.32; *p* = 0.128), reflecting only 11 events. This sensitivity result should therefore be regarded as supportive of caution rather than evidence against or for a direct causal effect.

These findings describe prognosis under the observed care pathway rather than the natural history of untreated Doppler abnormalities. The median interval from index assessment to delivery was similar in the normal- and abnormal-Doppler groups (18 vs. 16 days), yet fetal/placental indications for delivery were substantially more frequent with abnormal Doppler (35.9% vs. 16.6%). Earlier delivery can simultaneously prevent fetal deterioration and increase prematurity-related neonatal care requirements, as illustrated by TRUFFLE [10,22]. The attenuation of UA associations after conditional adjustment for gestational age at delivery is therefore compatible with management and prematurity contributing to NICU and respiratory outcomes, but it cannot be interpreted as a causal mediation estimate because delivery timing is post-index and potentially both a mediator and a collider.

### 4.7. Gestational Age and Phenotyping

Because the study was anchored to the index assessment, the 32-week strata represent gestational age at examination rather than a definitive classification of early- and late-onset FGR. Accurate onset classification would require longitudinal determination of the first assessment meeting diagnostic criteria and, where applicable, uterine artery Doppler information.

### 4.8. Strengths, Limitations and Research Implications

The strengths of this study include a clearly defined principal cohort, component-based composite outcome, mutually exclusive UA categories, and assessment of cross-validated discrimination and calibration. Several limitations should be acknowledged. The retrospective single-center design introduces potential selection bias and limits generalizability. Severe UA flow abnormalities were uncommon, leading to imprecise estimates for AEDF/REDF. Components of the composite outcome may overlap clinically, and NICU admission thresholds can vary by institution. The 32-week subgrouping reflects timing of the index examination rather than definitive onset of FGR. Uterine artery Doppler measurements were not routinely available in the retrospective records and could therefore not be incorporated into phenotype classification or prognostic modeling. This prevented assessment of the early-FGR diagnostic pathway combining EFW or AC below the 10th percentile with uterine artery PI above the 95th percentile. Other diagnostic pathways based on severe smallness or UA abnormalities remained assessable; however, comprehensive phenotypic stratification was limited by the available retrospective information. Consequently, our biometric cohort and index-assessment strata should not be regarded as equivalent to fully characterized Delphi-defined FGR populations. Comparisons with studies using comprehensive Doppler assessment and longitudinal diagnostic classification should therefore account for differences in cohort composition and phenotype ascertainment. Riley et al. emphasized that prediction-model sample size should account for event frequency, model complexity, and anticipated overfitting rather than relying on a simple fixed events-per-variable rule [21]. External validation and clinical-utility assessment are needed before the model can be considered for clinical implementation.

Additional limitations remain despite the supplementary analyses. Delivery indication categories and surveillance intensity were reconstructed retrospectively and may be subject to documentation variability. Component-specific NICU and respiratory-support models are vulnerable to management-related confounding, and conditioning on gestational age at delivery does not isolate causal effects. The hard-outcome composite contained only 11 events, precluding reliable multivariable modeling. Repeated cross-validation and refit-aware bootstrap improve internal robustness but do not replace external validation. The expanded baseline increases parameter burden and should be regarded as a sensitivity analysis rather than a preferred final model. Finally, decision-curve analysis and external calibration were not performed; improved discrimination should not be equated with demonstrated clinical benefit.

## 5. Conclusions

In pregnancies with suspected fetal smallness defined by EFW or AC below the 10th percentile, incorporation of fetal Doppler variables modestly improved internally validated discrimination beyond maternal and biometric characteristics. Abnormal UA Doppler, particularly AEDF/REDF, was associated with the composite adverse perinatal outcome. These associations should be interpreted cautiously because the composite was largely influenced by NICU admission and respiratory support, which may reflect both fetal condition and morbidity associated with delivery timing and clinical management. The findings support further investigation of integrated biometric and Doppler assessment; however, the present model requires external validation and assessment of clinical utility before clinical implementation.

## Figures and Tables

**Table 1:** Cohort characteristics and outcome distribution.

Population/Check	*n* (% of 606)	Composite Outcome	Interpretation
Full cohort	606 (100.0%)	151 (24.9%)	All study records
Biometric candidate (EFW or AC < 10th)	429 (70.8%)	111 (25.9%)	Prespecified main comparison sample
Neither size nor Doppler flag	143 (23.6%)	24 (16.8%)	Outside prespecified screening criteria
Doppler only; no small biometry	34 (5.6%)	16 (47.1%)	Separate exploratory category
Expanded suspected-growth sample	463 (76.4%)	127 (27.4%)	Alternative sensitivity sample

Note: Data are presented as *n* (%) or mean ± standard deviation, as appropriate. EFW, estimated fetal weight; AC, abdominal circumference. The primary adverse perinatal composite was defined as at least one of the prespecified neonatal or perinatal outcome components.

**Table 2:** Maternal and obstetric characteristics.

Variable	All (*n* = 429)	No (*n* = 318)	Yes (*n* = 111)	*p*
Maternal age, years	28.43 ± 5.61	28.30 ± 5.63	28.81 ± 5.58	0.330
Gravidity	2.79 ± 1.23	2.81 ± 1.24	2.71 ± 1.21	0.531
Parity	0.55 ± 0.69	0.57 ± 0.68	0.50 ± 0.74	0.187
Prior abortions	0.37 ± 0.65	0.40 ± 0.69	0.28 ± 0.51	0.211
Index gestational age, weeks	33.07 ± 3.10	33.52 ± 2.99	31.79 ± 3.06	<0.001
Regular antenatal follow-up	330 (76.9%)	235 (73.9%)	95 (85.6%)	0.012
Smoking	58 (13.5%)	38 (11.9%)	20 (18.0%)	0.107
Chronic hypertension	33 (7.7%)	26 (8.2%)	7 (6.3%)	0.524
Pregestational diabetes	15 (3.5%)	13 (4.1%)	2 (1.8%)	0.373
Gestational diabetes	38 (8.9%)	28 (8.8%)	10 (9.0%)	0.948
Previous FGR	42 (9.8%)	32 (10.1%)	10 (9.0%)	0.748
Previous preeclampsia	35 (8.2%)	23 (7.2%)	12 (10.8%)	0.236
Previous stillbirth	14 (3.3%)	8 (2.5%)	6 (5.4%)	0.209
Aspirin use	101 (23.5%)	73 (23.0%)	28 (25.2%)	0.628

Note: Data are presented as mean ± standard deviation or *n* (%), as appropriate. FGR, fetal growth restriction. *p* values refer to comparisons between pregnancies without and with the adverse perinatal composite.

**Table 3:** Fetal biometric indices.

Variable	All (*n* = 429)	No (*n* = 318)	Yes (*n* = 111)	*p*
EFW percentile	9.05 ± 6.99	9.53 ± 7.12	7.68 ± 6.44	0.022
AC percentile	7.06 ± 6.35	7.03 ± 6.46	7.14 ± 6.03	0.691
Estimated fetal weight, g	1933.87 ± 858.07	2067.25 ± 884.78	1551.77 ± 640.36	<0.001
Abdominal circumference, mm	267.95 ± 34.01	272.00 ± 33.04	256.33 ± 34.21	<0.001
Index gestational age, weeks	33.07 ± 3.10	33.52 ± 2.99	31.79 ± 3.06	<0.001
EFW < 3rd percentile	116 (27.0%)	80 (25.2%)	36 (32.4%)	0.137
AC < 3rd percentile	146 (34.0%)	109 (34.3%)	37 (33.3%)	0.857
EFW < 10th percentile	275 (64.1%)	194 (61.0%)	81 (73.0%)	0.024
AC < 10th percentile	342 (79.7%)	256 (80.5%)	86 (77.5%)	0.495

Note: Data are presented as mean ± standard deviation or *n* (%), as appropriate. EFW, estimated fetal weight; AC, abdominal circumference. Percentile thresholds refer to the fetal growth reference used in the study.

**Table 4:** Fetal Doppler characteristics.

Variable	All (*n* = 429)	No (*n* = 318)	Yes (*n* = 111)	*p*
Umbilical artery PI	1.17 ± 0.21	1.14 ± 0.19	1.26 ± 0.22	<0.001
Middle cerebral artery PI	1.59 ± 0.21	1.62 ± 0.21	1.52 ± 0.18	<0.001
Cerebroplacental ratio	1.41 ± 0.33	1.47 ± 0.33	1.24 ± 0.28	<0.001
UA-PI > 95th percentile	163 (38.0%)	97 (30.5%)	66 (59.5%)	<0.001
AEDF	17 (4.0%)	7 (2.2%)	10 (9.0%)	0.003
REDF	2 (0.5%)	1 (0.3%)	1 (0.9%)	0.451
MCA-PI < 5th percentile	63 (14.7%)	44 (13.8%)	19 (17.1%)	0.401
Abnormal CPR	95 (22.1%)	59 (18.6%)	36 (32.4%)	0.002
Abnormal ductus venosus	5 (1.2%)	1 (0.3%)	4 (3.6%)	0.017
Any abnormal fetal Doppler	206 (48.0%)	121 (38.1%)	85 (76.6%)	<0.001
UA: normal PI and forward flow	266 (62.0%)	221 (69.5%)	45 (40.5%)	<0.001
UA: elevated PI and forward flow	144 (33.6%)	89 (28.0%)	55 (49.5%)	<0.001
UA: absent/reversed flow	19 (4.4%)	8 (2.5%)	11 (9.9%)	0.002

Note: Data are presented as mean ± standard deviation or *n* (%), as appropriate. UA, umbilical artery; PI, pulsatility index; MCA, middle cerebral artery; CPR, cerebroplacental ratio; AEDF, absent end-diastolic flow; REDF, reversed end-diastolic flow. UA categories are mutually exclusive.

**Table 5:** Maternal complications and management.

Variable	All (*n* = 429)	No (*n* = 318)	Yes (*n* = 111)	*p*
Preeclampsia	47 (11.0%)	33 (10.4%)	14 (12.6%)	0.516
Preeclampsia with severe features	19 (4.4%)	12 (3.8%)	7 (6.3%)	0.286
Gestational hypertension	39 (9.1%)	31 (9.7%)	8 (7.2%)	0.423
HELLP syndrome	4 (0.9%)	2 (0.6%)	2 (1.8%)	0.276
PPROM	26 (6.1%)	16 (5.0%)	10 (9.0%)	0.131
Preterm labor	76 (17.7%)	45 (14.2%)	31 (27.9%)	0.001
Antenatal corticosteroids	230 (53.6%)	163 (51.3%)	67 (60.4%)	0.098
Magnesium sulfate	26 (6.1%)	15 (4.7%)	11 (9.9%)	0.048
Antenatal hospitalization	126 (29.4%)	95 (29.9%)	31 (27.9%)	0.698
Cesarean delivery	244 (56.9%)	172 (54.1%)	72 (64.9%)	0.048
Cesarean for fetal distress	55 (12.8%)	35 (11.0%)	20 (18.0%)	0.057

Note: Data are presented as *n* (%). HELLP, hemolysis, elevated liver enzymes and low platelet count; PPROM, preterm prelabor rupture of membranes. *p* values are two-sided.

**Table 6:** Delivery and neonatal outcomes by Doppler.

Variable	All (*n* = 429)	Normal (*n* = 223)	Abnormal (*n* = 206)	*p*
Gestational age at delivery, weeks	35.81 ± 2.71	36.50 ± 2.62	35.06 ± 2.60	<0.001
Birth weight, g	2295.05 ± 705.40	2474.72 ± 670.64	2100.56 ± 691.87	<0.001
5-min Apgar	8.88 ± 0.81	8.93 ± 0.76	8.83 ± 0.87	0.320
Umbilical artery pH	7.26 ± 0.05	7.26 ± 0.05	7.26 ± 0.06	0.772
5-min Apgar < 7	4 (0.9%)	1 (0.4%)	3 (1.5%)	0.355
Umbilical artery pH < 7.10	5 (1.2%)	1 (0.4%)	4 (1.9%)	0.199
Delivery < 37 weeks	272 (63.4%)	108 (48.4%)	164 (79.6%)	<0.001
Delivery < 34 weeks	89 (20.7%)	34 (15.2%)	55 (26.7%)	0.003
Delivery < 32 weeks	33 (7.7%)	12 (5.4%)	21 (10.2%)	0.062
NICU admission	105 (24.5%)	25 (11.2%)	80 (38.8%)	<0.001
Respiratory support	69 (16.1%)	18 (8.1%)	51 (24.8%)	<0.001
Mechanical ventilation	23 (5.4%)	6 (2.7%)	17 (8.3%)	0.011
Neonatal hypoglycemia	48 (11.2%)	19 (8.5%)	29 (14.1%)	0.068
Neonatal sepsis	19 (4.4%)	8 (3.6%)	11 (5.3%)	0.378
Perinatal death	2 (0.5%)	1 (0.4%)	1 (0.5%)	1.000
Hard adverse-outcome sensitivity composite	11 (2.6%)	3 (1.3%)	8 (3.9%)	0.128
Composite adverse perinatal outcome	111 (25.9%)	26 (11.7%)	85 (41.3%)	<0.001

Note: Data are presented as mean ± standard deviation or *n* (%), as appropriate. NICU, neonatal intensive care unit. The adverse perinatal composite was counted once per pregnancy when at least one component was present. Respiratory support denotes documented neonatal assistance beyond room air, including supplemental oxygen, high-flow nasal cannula, continuous positive airway pressure (CPAP)/bilevel positive airway pressure (BiPAP), or invasive mechanical ventilation. The hard sensitivity composite comprised five-minute Apgar < 7, umbilical artery pH < 7.10, or perinatal death; its *p* value is from Fisher’s exact test.

**Table 7:** Index gestational age–Doppler strata.

Index-Time Stratum × Doppler	*n*	Composite Outcome	NICU	Birth < 34 Weeks	Delivery Weeks
<32 weeks, Doppler normal	61	12 (19.7%)	11 (18.0%)	31 (50.8%)	33.69 ± 2.66
<32 weeks, Doppler abnormal	71	37 (52.1%)	34 (47.9%)	48 (67.6%)	32.71 ± 2.45
≥32 weeks, Doppler normal	162	14 (8.6%)	14 (8.6%)	3 (1.9%)	37.56 ± 1.66
≥32 weeks, Doppler abnormal	135	48 (35.6%)	46 (34.1%)	7 (5.2%)	36.29 ± 1.66
Overall four-group comparison	429	Pearson χ^2^ *p* < 0.001	—	—	—

Note: Gestational-age strata refer to the timing of the index assessment (<32 vs. ≥32 weeks) and should not be interpreted as independently adjudicated early- and late-onset FGR phenotypes. NICU, neonatal intensive care unit.

**Table 8:** Cohort-restriction sensitivity.

Sample	*n*	Composite Outcome Rate	Doppler Abnormal	Doppler Normal	Crude OR (95% CI)	*p*
All evaluated pregnancies	606	151 (24.9%)	101/240 (42.1%)	50/366 (13.7%)	4.59 (3.10–6.80)	<0.001
Biometric candidate (main)	429	111 (25.9%)	85/206 (41.3%)	26/223 (11.7%)	5.32 (3.25–8.72)	<0.001
Excluding neither-criterion group	463	127 (27.4%)	101/240 (42.1%)	26/223 (11.7%)	5.51 (3.40–8.92)	<0.001
Index examination < 32 weeks within principal cohort	132	49 (37.1%)	37/71 (52.1%)	12/61 (19.7%)	4.44 (2.03–9.74)	<0.001
Index examination ≥ 32 weeks within principal cohort	297	62 (20.9%)	48/135 (35.6%)	14/162 (8.6%)	5.83 (3.04–11.19)	<0.001

Note: ORs compare abnormal versus normal Doppler status within each specified cohort. OR, odds ratio; CI, confidence interval. Sensitivity analyses involve overlapping populations and are therefore not independent comparisons.

## Data Availability

The datasets generated and/or analyzed during the current study are not publicly available due to institutional data protection policies and patient confidentiality considerations. De-identified data may be made available from the corresponding author upon reasonable request and with permission from the relevant institution.

## Supplementary Materials

The following supporting information can be downloaded at: https://www.mdpi.com/article/10.3390/biomedicines14102370/s1, Table S1: Primary-composite overlap and component-specific adjusted analyses. Table S2: Management-pathway descriptors by Doppler category. Table S3: Alternative Doppler specifications using identical cross-validation folds. Table S4: Expanded clinically justified baseline sensitivity model. Table S5: Complete principal-model coefficients and intercepts. Table S6: Calibration by predicted-risk decile.
