# Supplementary material for: Incremental Prognostic Value of Fetal Doppler Beyond Biometry for Adverse Perinatal Outcomes in Suspected Fetal Growth Restriction: A Retrospective Cohort Study
Source: Biomedicines. 2026 Oct 17;14(10):2370. doi: 10.3390/biomedicines14102370 (PMC13702095; doi:10.3390/biomedicines14102370)
Supplement: Supplementary file 1 [file biomedicines-14-02370-s001.zip › biomedicines-4616154-supplementary.pdf]

Incremental Prognostic Value of Fetal Doppler Beyond Biometry for Adverse Perinatal Outcomes in Suspected Fetal Growth Restriction: A Retrospective Cohort Study

Supplementary analyses

Supplementary Table S1. Primary-composite overlap and component-specific adjusted analyses.

Component configuration	n	% of 111 composite-positive
NICU + respiratory support	66	59.5
NICU without respiratory support	39	35.1
Respiratory support without NICU	3	2.7
Other composite component(s) only	3	2.7
Total	111	100.0

NICU, neonatal intensive care unit. Mutually exclusive overlap groups sum to the 111 primary-composite events.

Predictor	NICU aOR (95% CI); p	Respiratory support aOR (95% CI); p	NICU + GA-delivery aOR	Respiratory + GA-delivery aOR
UA high PI, forward flow	2.54 (1.51–4.27); <0.001	2.20 (1.23–3.94); 0.008	1.68 (0.95–2.96)	1.54 (0.80–2.95)
UA AEDF/REDF	6.12 (2.20–17.02); 0.001	5.43 (1.82–16.18); 0.002	4.12 (1.36–12.48)	3.71 (1.13–12.19)
MCA-PI <5th percentile	1.09 (0.56–2.11); 0.803	1.18 (0.56–2.51); 0.663	1.07 (0.54–2.14)	1.14 (0.52–2.49)
GA at delivery, per week	-	-	0.58 (0.50–0.67)	0.56 (0.47–0.66)
OOF AUC, baseline → Doppler	0.650 → 0.699	0.654 → 0.700	-	-
OOF Brier, baseline → Doppler	0.174 → 0.165	0.132 → 0.126	-	-

All antenatal component-specific models use maternal age, index gestational age, EFW percentile, AC percentile, chronic hypertension, mutually exclusive UA categories, and abnormal MCA-PI. Models including gestational age at delivery are conditional sensitivity analyses, not antenatal prediction models.

Supplementary Table S2. Management-pathway descriptors by Doppler category.

Descriptor	Normal Doppler (n=223)	Abnormal Doppler (n=206)	p
Index-to-delivery interval, days, median (IQR)	18 (9–31)	16 (7–29)	0.148
Fetal/placental delivery indication	37 (16.6%)	74 (35.9%)	<0.001
Maternal delivery indication	24 (10.8%)	27 (13.1%)	

Spontaneous labor/PPROM	60 (26.9%)	52 (25.2%)	
Planned/other obstetric indication	102 (45.7%)	53 (25.7%)	
Total	223 (100%)	206 (100%)	

The p value for delivery indication is the global comparison across the four mutually exclusive categories. Indications are post-index descriptors and are not included in antenatal prediction models.

Supplementary Table S3. Alternative Doppler specifications using identical cross-validation folds

Model	OOF AUC	OOF Brier	Calibration intercept	Calibration slope	ΔAUC vs baseline (95% CI)
Baseline	0.642	0.183	−0.169	0.829	Reference
Baseline + UA categories	0.679	0.177	−0.154	0.836	+0.037 (0.002–0.074)
Baseline + continuous CPR	0.684	0.176	−0.151	0.839	+0.042 (0.004–0.081)
Baseline + UA categories + abnormal MCA-PI	0.690	0.174	−0.147	0.841	+0.049 (0.005–0.094)

Direct paired ΔAUCs: integrated vs UA-only +0.011 (95% CI −0.012 to 0.034); integrated vs CPR +0.006 (−0.014 to 0.026); CPR vs UA-only +0.005 (−0.019 to 0.029).

Validation metric	Baseline	Integrated Doppler	Increment / improvement
50×10-fold CV median AUC	0.643	0.689	0.046 (5th–95th: 0.031–0.061)
50×10-fold CV median Brier	0.183	0.174	0.009 (5th–95th: 0.004–0.014)
50×10-fold median calibration intercept	−0.166	−0.144	-
50×10-fold median calibration slope	0.832	0.846	Integrated 5th–95th: 0.786–0.914
Refit-bootstrap optimism-corrected AUC	0.645 (0.589–0.697)	0.687 (0.635–0.736)	0.042 (0.007–0.078)
Refit-bootstrap optimism-corrected Brier	0.182	0.175	0.007 (0.001–0.014)

Supplementary Table S4. Expanded clinically justified baseline sensitivity model

Metric	Expanded baseline	Expanded baseline + Doppler
Predictors	Age; index GA; EFW percentile; AC percentile; chronic hypertension; smoking; pregestational diabetes; previous FGR; previous preeclampsia	Expanded baseline + UA categories + MCA-PI <5th percentile
OOF AUC	0.656	0.695
OOF Brier	0.181	0.173
Calibration intercept	−0.181	−0.159
Calibration slope	0.814	0.829
Paired ΔAUC	Reference	+0.039 (95% CI 0.003–0.078)
Brier improvement	Reference	+0.008 (95% CI −0.001 to 0.016)

Supplementary Table S5. Complete principal-model coefficients and intercepts

Term	Baseline β	Baseline aOR (95% CI); p	Integrated β	Integrated aOR (95% CI); p
Intercept	4.533	-	3.451	-
Maternal age, per year	0.020	1.02 (0.97–1.06); 0.466	0.020	1.02 (0.98–1.06); 0.360
Index gestational age, per week	-0.174	0.84 (0.78–0.90); <0.001	-0.163	0.85 (0.78–0.91); <0.001
EFW percentile, per centile	-0.041	0.96 (0.93–1.00); 0.038	-0.020	0.98 (0.95–1.02); 0.386
AC percentile, per centile	0.000	1.00 (0.96–1.03); 0.830	0.010	1.01 (0.97–1.05); 0.639
Chronic hypertension	-0.174	0.84 (0.34–2.06); 0.704	-0.051	0.95 (0.38–2.37); 0.920
UA high PI, forward flow	-	-	0.959	2.61 (1.58–4.32); <0.001
UA AEDF/REDF	-	-	1.887	6.60 (2.40–18.18); <0.001
MCA-PI <5th percentile	-	-	0.131	1.14 (0.60–2.14); 0.690

β coefficients for non-intercept terms are the natural logarithms of the reported adjusted odds ratios. The reported intercepts are the fitted-model intercepts.

Supplementary Table S6. Calibration by predicted-risk decile

Decile	n	Baseline mean predicted	Baseline observed n/N (%)	Integrated mean predicted	Integrated observed n/N (%)
1	43	0.070	3/43 (7.0%)	0.040	2/43 (4.7%)
2	43	0.100	4/43 (9.3%)	0.070	3/43 (7.0%)
3	43	0.130	5/43 (11.6%)	0.100	4/43 (9.3%)
4	43	0.160	7/43 (16.3%)	0.140	5/43 (11.6%)
5	43	0.200	8/43 (18.6%)	0.180	7/43 (16.3%)
6	43	0.240	10/43 (23.3%)	0.230	9/43 (20.9%)
7	43	0.290	12/43 (27.9%)	0.290	12/43 (27.9%)
8	43	0.350	14/43 (32.6%)	0.360	16/43 (37.2%)
9	43	0.420	20/43 (46.5%)	0.470	22/43 (51.2%)
10	42	0.540	28/42 (66.7%)	0.660	31/42 (73.8%)

Each decile contains 42–43 pregnancies. The associated Figure 2 displays 95% Wilson confidence intervals around the observed proportions.
